# Role of Netrin-1 in staging hypoxic ischemic encephalopathy

**DOI:** 10.1590/1806-9282.20241889

**Published:** 2025-06-16

**Authors:** Nur Aycan, Derya Çay Demir, Eyyüp Yürektürk, Murat Başaranoglu, Serap Karaman, Oğuz Tuncer

**Affiliations:** 1 Van Yuzuncu Yil University, Department of Pediatrics – Van, Türkiye.; 2 Van Yuzuncu Yil University, Department of Chemistry – Van, Türkiye.; 3 Van Yuzuncu Yil University, Department of Neonatology – Van, Türkiye.

**Keywords:** Netrin-1, Term birth, Hypoxic-ischemic encephalopathy, Biomarker

## Abstract

**OBJECTIVE:**

The diagnosis and prognosis of neonatal hypoxic-ischemic encephalopathy are established through clinical evidence and laboratory, imaging, and electrophysiological assessments of the nervous system. Netrin-1 was the first axon guidance molecule identified as a critical component of embryonic development in vertebrates and has a solid chemotropic function for angiogenesis, morphogenesis, cell migration, and axonal guidance. It was hypothesized that Netrin-1 will differ at different hypoxic-ischemic encephalopathy stages.

**METHODS:**

This study included 75 hospitalized hypoxic-ischemic encephalopathy newborns and 48 healthy newborns born at the same hospital and followed up only by their mothers. Demographic, laboratory, and Netrin-1 data were evaluated for all hypoxic-ischemic encephalopathy stages.

**RESULTS:**

Serum Netrin-1 concentrations were significantly greater in patients with moderate and severe hypoxic-ischemic encephalopathy who underwent therapeutic hypothermia than in controls and patients with severe hypoxic-ischemic encephalopathy. However, serum Netrin-1 concentrations were not significantly greater in patients with mild hypoxic-ischemic encephalopathy than in controls. In 75 hypoxic-ischemic encephalopathy patients, correlations of Netrin-1 with lactate, uric acid, and lactate dehydrogenase were statistically significant (p=0.0001, 0.008, and 0.043, respectively).

**CONCLUSION:**

Netrin-1 significantly increased in moderate and severe patients. Therefore, this marker could be a biomarker for staging hypoxic-ischemic encephalopathy and therapeutic hypothermia and predicting the prognosis of neonatal hypoxic-ischemic encephalopathy patients.

## INTRODUCTION

Perinatal asphyxia is marked by a disruption in the exchange of respiratory gases (oxygen and carbon dioxide), leading to decreased oxygen levels (hypoxemia) and increased carbon dioxide levels (hypercapnia), often accompanied by metabolic acidosis^1^. Hypoxic-ischemic encephalopathy (HIE) is a brain injury or damage resulting from decreased oxygen in the brain during the perinatal period^2^. In developing nations, the incidence (10–20/1,000 live births) is generally greater than that in the developed world (2–3/1,000 live births)^3^. Neonatal HIE diagnosis and prognosis are established through clinical evidence, laboratory measurements, imaging scans, and electrophysiological assessments of the nervous system^4,5^. Employing biomarkers to monitor brain damage and evaluate the effectiveness of neuroprotective measures could facilitate prompt intervention and management of neonatal HIE, potentially reducing mortality rates^6^.

Netrin-1 (NT-1) is a well-characterized protein of the netrin protein family. NT-1 is evolutionarily related to the extracellular matrix protein laminin^7^ and abundantly expressed within neurons and facilitates axon outgrowth and neuronal migration during embryonic maturation^8^. NT-1 is the initial regulator of axon guidance and plays a fundamental role in chemotropism, morphogenesis, angiogenesis, cell migration, and axonal guidance^7,9–12^. Serum NT-1 concentrations are independently associated with poor outcomes in adult traumatic brain injury (TBI) patients^13^, predicting cognitive impairment after spinal cord injury^14^, and are a promising target for treating ischemic stroke^10,15^.

The objective of the study was to evaluate whether this substance, which is thought to play a role in many neurologic diseases, differs in relation to the severity of perinatal HIE and to determine its relationship with altered laboratory values in HIE.

## METHODS

The study included 75 infants with HIE with a gestational age (GA) of ≥37 weeks who were hospitalized at Yuzuncu Yil University between January and December 2023 and 48 healthy infants born at the same hospital who were followed up only by their mothers as the control group. The study group included HIE newborns with GA ≥37 weeks who were admitted to the neonatal intensive care unit (NICU) with a diagnosis of perinatal hypoxia. The diagnosis of perinatal hypoxia was based on the presence of an acute intrapartum/peripartum event with pH ≥7.00 or base deficit ≥12 mmol/L in the first hour after birth or blood gas from cord blood, Apgar score <5 at the 5th–10th min, or the need for prolonged resuscitation. Neonates with GA <37 weeks, congenital anomalies, congenital heart defects, cerebral anomalies, genetic diseases, sepsis, or no parental approval were excluded from the study.

Sarnat stage was categorized as HIE, and stages I–III indicated mild, moderate, and severe HIE, respectively^16^. In a tertiary care NICU where ~1,000 newborn babies are treated annually, the sample size was generated by including all babies hospitalized due to HIE who met the inclusion criteria in the specified date range after obtaining ethics committee approval under the guidelines of the NICU protocol. The study received approval from the local ethics committee before commencement (Yuzuncu Yil University Clinical Research Ethics Committee date no. 2022/12/28-01) and was conducted in accordance with the principles outlined in the Declaration of Helsinki. Before the research, the parents of the infants participating in the study were informed about the project and conduct of the study. The study flow was designed accordingly, consent was obtained, and blood samples were collected from all patients and healthy newborns.

The whole-body cooling procedure involved babies with eligibility criteria, including pH <7.0 or base deficit ≥16 mmol/L in an umbilical cord blood sample or any blood during the first hour after birth, an acute perinatal event, and either a 10-min Apgar score ≤5 or assisted ventilation initiated at birth and persisting for at least 10 min. Hypothermia induction was achieved using the Cool Star (CSM2020013-GMS Medical, Turkey) device to maintain a rectal temperature between 33.5 and 34.5°C for 72 h. This was followed by a rewarming event that allowed the body to reach normal body temperature in 6 h^17^.

The demographic characteristics; 1st-, 5th-, and 10th-min Apgar scores; resuscitation in the delivery room; seizure status; amplitude-integrated electroencephalography (aEEG; Natus-Olympic Brainz Monitor); blood gas, liver, and kidney function tests; complete blood count; C-reactive protein; coagulopathy tests; mortality; and length of hospital stay were recorded. All blood values and NT-1 were obtained at the first admission to the NICU from blood drawn at the same time before hypothermia treatment was started (<6 h). Resuscitation required positive pressure ventilation in the delivery room for at least 30 s^18,19^.

Blood sampling for NT-1 was performed before the onset of hypothermia and timed at a median of 88.5 min postpartum (interquartile range=61.25–120). Venous blood (2 mL) from each patient and control group was collected in a transparent nonanticoagulant tube and centrifuged at 4,000 rpm for 15 min after 20 min coagulation. The obtained sera were stored at −80°C until the day of the study. Serum NT-1 was measured on all samples on the same day by a single investigator blinded to the participants using a commercial enzyme-linked immunosorbent assay (ELISA) (human Ntn1 ELISA kit, catalog no. E-EL-H2328, product size 96T/48T/24T/96T*5) according to the manufacturer's instructions. The optical densities of the samples were compared to the standard curve, and the human NT-1 concentration was calculated and expressed as pg/mL.

### Statistical analysis

Descriptive statistics for continuous variables were expressed as mean, standard error, median, minimum, and maximum values, whereas descriptive statistics for categorical variables were expressed as numbers and percentages. One-way analysis of variance or Kruskal-Wallis test was used to compare continuous variables according to categorical variables. Mann-Whitney U test was used for pairwise comparisons. Post hoc analyses were conducted using Duncan's test. Pearson's correlation coefficients were calculated to determine the relationships between continuous variables. The χ^2^ test was used to determine the relationships between categorical variables, and ratio comparisons were made when necessary. Receiver operating characteristic curve (ROC) analysis was performed to detect the predictive ability and determine the cutoff value. The statistical significance level was set at 0.05, and the SPSS (IBM-version 26) statistical package program was used for calculations.

## RESULTS

During the specified study period, 75 HIE patients were included because 11 of 86 patients were not included for not meeting the inclusion criteria due to congenital anomalies. A total of 48 healthy neonates born at the same hospital during the same period were included in the control group. Among the HIE neonates, 22 (29.3%) were born at a university hospital and 53 (70.3%) were referred from nearby hospitals. The time to start therapeutic hypothermia treatment according to Sarnat staging and hypothermia treatment initiation criteria (n=62) was 111.26±7.09 min (min–max: 25–310).

The body weight (BW), sex, type of delivery, maternal age, GA, gestational diabetes status, and consanguineous marriage status did not differ between the groups (p=0.09, p=0.58, p=0.63, p=0.89, p=0.21, p=0.61, and p=0.55, respectively). Apgar scores at the 1st, 5th, and 10th min were statistically significantly lower in HIE patients (p=0.0001, p=0.0001 and p=0.0001, respectively). When NT-1 levels between the sexes of hypoxia patients were compared, no statistically significant difference was found between female (85.5±11.34) and male (80.72±12.05) infants (p>0.05).

Initial blood gas results and clinical features of patients with Sarnat stage I/II/III HIE are reported in Table 1.

**Table 1:** Initial blood gas results and clinical features of patients with Sarnat stage I/II/III hypoxic-ischemic encephalopathy

		Stage I (n=13)	Stage II (n=40)	Stage III (n=22)	p-value
Initial pH*		7.09 (0.01)^a^	6.99 (0.009)^b^	6.97 (0.01)^b^	0.0001
Initial base excess* (mEq/La)		11.8 (0.71)^a^	16.9 (0.3)^b^	15.7 (1.6)^b^	0.0001
Lactate in blood gas* (mmol/L)		5.44 (0.4)^a^	7.55 (0.21)^b^	11.11 (0.63)^c^	0.0001
Initial HCO_3_in blood gas* (mmol/L)		15.92 (0.7)^a^	16.01 (2.03)^a^	13.89 (0.7)^b^	0.02
Clinical seizures (n)		–	15 (37.5%)	15 (68.2%)	0.02
aEEG	Normal trace	11 (84.6%)	24 (60%)	2 (9%)	0.0001
	Abnormal trace	2 (15.4%)	16 (40%)	8 (36%)	
	Burst pattern	–	–	12 (54%)	
Inotropic support(n)		2 (15.4%)	4 (10%)	13 (59.1%)	0.0001
Days in hospital**		9 (7–12)^a^	9 (6–14)^a^	13 (8–38)^b^	0.0001

* mean (SEM),

** median (min–max).

pH: potential of hydrogen, HCO_3_: sodium bicarbonate; aEEG: amplitude-integrated electroencephalography. In pairwise comparisons, different letters show statistical significance, while the same letters are not statistically significant.

Magnetic resonance imaging (MRI) was performed after hypothermia treatment at a median age of 5 days, and the results were available for all 75 patients in the HIE group. A total of 33 (44%) neonates (11 with moderate HIE and 22 with severe HIE) with abnormal MRI results exhibited diffuse white matter injury and watershed infarcts, and 3 HIE newborns had basal ganglia injuries. All babies (n=4) who died were in Sarnat stage III and had infarcts on MRI.

Table 2 compares the hematologic and biochemical laboratory results between healthy newborns and patients with different hypoxia stages. Serum NT-1 measurements were statistically significantly greater in HIE neonates. The cutoff point for NT-1 was 39.1285 pg/mL in the moderate/severe HIE neonates after ROC analysis; the area under the curve was 0.818 (p<0.001) (Table 3).

**Table 2:** Laboratory results of healthy neonates and staged hypoxic-ischemic encephalopathy patients

	Healthy (n=48)	Stage I (n=13)	Stage II (n=40)	Stage III (n=22)	p-value
CRP (mg/L)	2.01 (0.007)^a^	2.45 (0.28)^a^	3.11 (0.36)^ab^	4.13 (0.6)^b^	0.0025
Glucose#	86.92 (2.61)^a^	78.76 (9.95)^a^	77.72 (4.90)^a^	67.59 (2.96)^a^	0.067
BUN (mg/dL)	7.64 (0.18)^a^	11.6 (0.74)^b^	11.87 (0.47)^b^	12.3 (0.54)^b^	0.0001
Creatinine##	0.6 (0.04)^a^	0.89 (0.05)^b^	0.87 (0.04)^b^	1.17 (0.05)^c^	0.0001
CK###	568.89 (35.7)^a^	1,537.2 (171)^b^	2,063.9 (186)^b^	2,060.7 (180)^b^	0.0001
Sodium#	137.92 (1.05)^a^	139.15 (1.65)^a^	134.47 (0.52)^b^	134 (0.52)^b^	0.0001
Potassium#	4.45 (0.09)^a^	5.10 (0.13)^b^	5.26 (0.07)^b^	5.37 (0.14)^b^	0.0001
Calcium#	9.36 (1.13)^c^	7.99 (0.29)^a^	8.44 (0.16)^ab^	9.02 (0.29)^bc^	0.0001
LDH###	361.64 (15.9)^a^	1,055.69 (94)^b^	1,346.65 (57)^c^	1,533.5 (94.8)^c^	0.0001
Uric acid##	4.8 (0.18)^a^	7.51 (0.3)^b^	8.17 (1.5)^bc^	8.86 (2.01)^c^	0.0001
AST###	94.64 (2.83)^a^	212.84 (38)^ab^	204.67 (20.2)^ab^	312.9 (125.6)^b^	0.0001
ALT###	28.28 (1.92)^a^	63.15 (8.5)^ab^	71.37 (5.87)^ab^	116.9 (42.21)^b^	0.016
INR	1.03 (0.01)^a^	1.45 (0.02)^b^	1.44 (0.02)^b^	1.61 (0.07)^c^	0.0001
aPTT (sn)	36.42 (0.58)^a^	43.93 (1.24)^b^	50.26 (1.68)^c^	54.5 (3.3)^c^	0.0001
PT (sn)	13.57 (0.3)^a^	26.05 (1.55)^b^	23.72 (0.93)^b^	25.87 (1.69)^b^	0.0001
WBC (10^9^/L)	10,812.5 (414)^a^	18,033 (1,292)^b^	19,870 (1,158)^b^	18,364 (1,459)^b^	0.0001
Neutr (10^9^/L)	6,687 (266)^a^	8,498 (655)^a^	11,927 (794)^b^	11,345 (1,224)^b^	0.0001
Neutr/lymph	2.5 (0.17)^b^	1.36 (0.17)^a^	2.36 (0.17)^b^	2.39 (0.29)^b^	0.015
RBC (10^12^/L)	4.81 (0.13)^a^	4.60 (0.24)^a^	5.05 (0.11)^a^	4.84 (0.12)^a^	0.21
Hgb (g/L)	17.67 (0.3)^a^	16.23 (0.4)^a^	16.98 (0.22)^a^	16.91 (0.41)^a^	0.053
Netrin-1¥ (pg/mL)	36.23 (5.9)^a^	32.13 (10.95)^a^	72.33 (8.4)^b^	131.88 (19)^c^	0.0001

CRP: C-reactive protein; BUN: blood urea nitrogen; CK: creatine kinase; LDH: lactate dehydrogenase; AST: aspartate aminotransferase; ALT: alanine aminotransferase; INR: international normalized ratio; aPTT: activated partial thromboplastin time; PT: prothrombin time; WBC: white blood cell; Neutr/lymph: neutrophil/lymphocyte; RBC: red blood cell; Hgb: hemoglobin.

# (mmol/L)

## (**μ**mol/L)

### (U/L) ANOVA: post-hoc: Duncan,

¥ Kruskal-Wallis, and Mann-Whitney U.

In pairwise comparisons, different letters show statistical significance, while the same letters are not statistically significant.

**Table 3:** The area under the curve, cutoff level, sensitivity, specificity, positive predictive value, and negative predictive value of serum lactate dehydrogenase, uric acid, and Netrin-1 for the moderate/severe vs. mild/control groups

	AUC	p-value	Cutoff level	Sensitivity (%)	Specificity (%)	PPV (%)	NPV (%)
LDH (U/L)	0.918	0.0001	898	87.4	91.4	83.7	97.2
Uric acid (μmol/L)	0.883	0.0001	6.75	82.9	82.4	90.3	76.7
Netrin-1 (pg/mL)	0.818	0.0002	39.12	81.8	89.4	74.7	96.2

PPV: positive predictive value, NPV: negative predictive value; AUC: area under the curve; LDH: lactate dehydrogenase.

Lactate was positively and closely correlated with serum NT-1 concentrations among HIE patients (r=0.509, p=0.0001). The correlation between uric acid and NT-1 was statistically significant (r=0.306, p=0.008). Lactate dehydrogenase (LDH) levels were positively correlated with NT-1 levels in HIE neonates (r=0.316, p=0.043). LDH, NT-1, and uric acid had high positive predictive values (PPVs) and negative predictive values (NPVs) in the moderate/severe vs. mild/control groups (Table 3). There was a statistically significant correlation between the time HIE babies spent in the hospital and the NT-1 level (r=0.441, p=0.0001).

## DISCUSSION

In this clinical research, serum NT-1 levels measured after the diagnosis of HIE according to defined criteria were higher in patients with moderate and severe HIE who received therapeutic hypothermia compared to controls and patients with mild HIE. In contrast, they were not significantly higher in patients with mild HIE than in controls.

In literature where different diagnostic or prognostic parameters (liver functions, renal functions, NT-1, neuron-specific enolase [NSE], lactate levels, imaging results, etc.) were studied in hypoxic infants, the mean birth weeks, mean BW, maternal age, sex distribution of the infants, and transfer mode (inborn/outborn) were similar to those in this study^19–22^. LDH is a good determinant of HIE during the first 12 h after birth, is of clinical interest in neonates with perinatal asphyxia, and potentially offers a safe prognostic marker^23^. Lee et al. reported that patients with mild HIE can be excluded using the combined biomarkers method, whereas those with moderate or severe neonatal HIE can be included, assisting in determining the optimal moment to initiate early hypothermia rescue treatment^20^.

In recent studies, the mechanism of encephalopathy has been evaluated for many brain-related biomarkers. S-100β and activin A are superior biomarkers for the early diagnosis of neonatal HIE^5^. Differences in the blood biomarkers of mild and moderate/severe HIE newborns were observed in terms of creatinine and white blood cell count^20^. In a study comparing the blood parameters of stage II/III patients with those of healthy newborns, in addition to low blood gas pH and HCO_3_, base excess (BE), blood urea nitrogen, creatinine, aspartate aminotransferase (AST), alanine aminotransferase (ALT), LDH, creatine kinase (CK), troponin I, and uric acid were significantly greater in stage II/III infants than in controls^22^.

NT-1 can inhibit glial fibrillary acidic protein expression, decrease chemokine secretion, and support recovery partly by reducing astrocyte activation, expanding the role of NT-1 in stroke pathology^10^. Under inflammatory conditions, blood–brain barrier endothelial cells strongly upregulate NT-1 levels in situ and in vitro, leading to endothelial cell activation and junctional breach^12^. The mean NT-1 concentration of patients in the appropriate GA group was significantly lower than that of the control group^11^. NSE and NT-1 parameters showed high specificity and sensitivity in moderate and severe infants to recommend therapeutic hypothermia^22^.

Serum NT-1 concentrations were significantly lower in moderate/severe TBI patients than in controls and were also significantly lower in severe TBI patients than in moderate and mild TBI patients^13^. In acute spinal canal injury, serum NT-1 levels are much lower, which is associated with decreased cognitive performance^14^. Evidence indicates that NT-1 may be a potential therapeutic candidate for decreasing stroke severity and improving patient prognosis. Its neuroprotective mechanisms may involve regulating apoptosis, angiogenesis, and neuroinflammation^15^. Moreover, NT-1 controlled programmed cell death mediated by the p53 gene in a rat ischemia stroke model^9^. Serum NT-1 concentrations were significantly associated with 90-day mortality, adverse outcomes, and overall survival in adult acute intracerebral hemorrhage patients^24^.

This study has some limitations as it was not multicenter and cord blood could not be obtained from every patient. Future studies are planned to evaluate patient outcomes and more parameters with more comprehensive studies.

Due to the severity of neonatal HIE, patients present different pathological changes in the brain; therefore, the expression of biomarkers also varies. Furthermore, no unified standard has been established between the severity of neonatal HIE brain injury and the concentration of relevant biomarkers in bodily fluids. This study will shed light on the role of NT-1 protein, which varies in different neurological diseases, in the treatment of "hypothermia" in newborns with HIE, as well as future steps toward developing diagnostic treatment for hypoxia.
